# Characterization and evaluation of the cytotoxic, antioxidant, and anti-human lung cancer properties of copper nanoparticles green-synthesized by fennel extract following the PI3K/AKT/Mtor signaling pathway

**DOI:** 10.1371/journal.pone.0309207

**Published:** 2025-01-09

**Authors:** Tao Huang, KaiLi Ma, Yihua Wang

**Affiliations:** Department of Oncology, Peking University First Hospital, Taiyuan Hospital, Taiyuan, Shanxi, China; Queen’s University Belfast, UNITED KINGDOM OF GREAT BRITAIN AND NORTHERN IRELAND

## Abstract

This work established the cytotoxic, antioxidant and anticancer effects of copper nanoparticles (CuNPs) manufactured with fennel extract, especially on non-small cell lung cancer (NSCLC) as well. CuNPs caused cytotoxicity in a dose-dependent manner for two NSCLC cell lines, A549 and H1650. At 100 μg/ml, CuNPs reduced cell viability to 70% in A549 cells and 65% in H1650 cells. which showed a cytotoxic effect (p<0. 05). Lactate dehydrogenase (LDH) was correspondingly present in a high proportion in the cells, demonstrated upon testing. Together with their cytotoxic properties, CuNPs demonstrated high antioxidative activity. When the concentration of the nano particles was high (100 μg/ml), the ratio of reactive oxygen species (ROS) was reduced as much as 50%, which in turn suggested antioxidant activity. There was plenty of evidence that CuNPs had anti-cancer potential; this has been shown by the effect of the molecules on the PI3K/AKT/mTOR pathway, which was one of the pathways crucial for cancer survival. Western blot analysis and qRT-PCR results indicated a widespread degradation of the proteins in this pathway upon CuNP exposure. Interestingly, there was a declined phosphorylation up to 75% of PI3K, AKT, and mTOR at 100 μg/ml (p<0. 001). In summary, these findings illustrated the mechanisms behind the therapeutic effect of CuNPs, thus making them good targets for the NSCLC treatment. CuNPs have cytotoxic and antioxidant capacity, as well as significant alterations in lung cancers pathway, and therefore they can be considered as anti-cancer candidates.

## Introduction

The biogenic synthesis of nanoparticles has gained significant attention due to its environmentally friendly and sustainable approach. Utilizing biological entities such as plants, bacteria, fungi, and algae for the synthesis of metallic nanoparticles offers several advantages, including the use of non-toxic chemicals, low energy requirements, and the potential for large-scale production. Recent advancements have demonstrated the effectiveness of biogenic nanoparticles in various biomedical applications, including cancer therapy, drug delivery, and antimicrobial treatments [1]. Moreover, plant-mediated synthesis offers a green chemistry approach, utilizing plant extracts rich in phytochemicals as reducing and stabilizing agents. This method is advantageous because it is simple, cost-effective, and scalable. Various plants have been successfully used to synthesize a wide range of nanoparticles, exhibiting significant biological activity [2]. The integration of biogenic nanoparticles in cancer therapy, particularly using the green synthesis approach, holds promising potential. These nanoparticles exhibit unique properties such as enhanced biocompatibility, stability, and biological activity, which are crucial for therapeutic applications. The biogenic synthesis not only aligns with sustainable development goals but also provides a versatile platform for producing nanoparticles with diverse biomedical applications [3].

Non-small cell lung cancer (NSCLC) is the leading type of cancer along with other cancers with the highest number of cancer related deaths in the world. On the other hand, being careful about the ineffective nature of conventional medical treatment and serious consequences caused by it makes sense to find an effective cure. Green nanoparticles from plant extracts, especially in nanotechnology innovations, have amazing advances in the studies of cancer. The green method of synthesis of copper nanoparticles (CuNPs) using a fennel extract obtained, possess outstanding cytotoxicity and antioxidant features [4]. These nanoparticles have a dual role in cancer therapy: inducing apoptosis and scavenging free radicals. The ability of CuNPs to trigger apoptosis in cancer cells is critical for their therapeutic potential, as it directly leads to the reduction of tumor cells. Concurrently, the antioxidant activity of CuNPs helps mitigate oxidative stress, which is often associated with inflammation and tumor progression. By reducing the levels of reactive oxygen species (ROS), CuNPs not only protect healthy cells but also enhance their therapeutic efficacy against cancer cells. Green cuNPs are obtained from organic compounds such as reduced copper salt which are gained from fennel; The size of the nanoparticles reduces, and their surface coating is obtained by the process. A key indicator which can further help in the discovery of their biological activities is the physiochemical properties of nanoparticles [5].

The morphology, composition and mineralization of CuNPs were found by employing TEM, SEM, XRD and DLS techniques. Nonetheless, the presence of the biomolecules oon the surface of CuNP has been corroborated by conducted Fourier transform infrared spectroscopy study (FTIR), implying that their potency as well as bioavailability may be enhanced. Along with the measurement of apoptosis using Annexin V-FITC and caspase activity, it can also be concluded that CuNPs significantly contribute to healthy cell proliferation. The ROS production, which may be the critical pathway, was investigated for their contribution to the toxicity of CuNPs [6].

Advantageously, despite their ability to induce oxidative stress, fennel extracted CuNPs are recognized as highly antioxidants. This behavior can be attributed to the bioactive compounds in fennel extract that are considered as CuNPs, providing the free radical scavenging abilities. This action is particularly important in cancer treatment and may reduce oxidative stress associated with inflammation and tumor growth. Investigating how CuNPs affect this process may shed light on their anti-inflammatory activities [7]. The testing continued to look at the expression of key proteins involved in cell cycle progression and apoptosis, such as cyclin D1, p21, and Bcl-2 family proteins. This article evaluated the synthesis, properties, and anticancer activity of CuNPs using fennel extraction and focused on their effects on human lung cancer via the PI3K/AKT/mTOR pathway [8]. Combining cytotoxic and antioxidant properties with studies on green plants, these nanoparticles offer an interesting guarantee a novel treatment approach in the fight against cancer.

## Materials and methods

### Ethical approval

In conducting the research on the effects of copper nanoparticles (CuNPs) on non-small-cell lung cancer (NSCLC) cell lines, strict adherence to ethical guidelines and compliance with established standards was maintained to ensure the integrity and ethical acceptability of the work. All experimental protocols involving human cell lines were rigorously reviewed and approved by the Institutional Review Board (IRB) at the host institution. The use of A549 and H1650 cell lines was conducted under strict laboratory conditions and was compliant with all relevant ethical regulations. Approval was granted after thorough review of the study’s aims, methods, and potential implications, ensuring that all experiments were conducted responsibly and ethically [9].

### Synthesis of copper nanoparticles

The synthesis of copper nanoparticles (CuNPs) using fennel extract involved several critical parameters, including the concentration of copper sulfate, the pH of the solution, the temperature, and the duration of the reaction. These parameters were optimized to achieve the desired particle size, stability, and yield of CuNPs. Copper sulfate pentahydrate (CuSO4·5H2O) and other chemicals were purchased from Sigma-Aldrich (analytical grade) and used without further purification. Fennel seeds were collected, air-dried to remove moisture, and ground into a fine powder using a mechanical grinder. The bioactive compounds were extracted from the powdered fennel seeds by maceration in ethanol (70% v/v) for 48 hours at room temperature, with intermittent shaking to ensure maximum yield. The extract was then filtered using Whatman No.1 filter paper and the solvent was evaporated under reduced pressure using a rotary evaporator at 40°C to obtain a concentrated extract. For the synthesis of copper nanoparticles, the concentrated fennel extract was mixed with an aqueous solution of copper sulfate under controlled conditions. The reaction mixture was maintained at a temperature of 60°C for 3 hours and stirred continuously at 400 rpm. To facilitate the reduction process, the pH of the solution was adjusted to 10 using sodium hydroxide (NaOH). The change in color of the solution from blue to dark brown indicated the formation of copper nanoparticles. The reaction was carried out for 3 hours to complete reduction of copper ions.

Then, the copper nanoparticles were isolated from the solution by centrifugation at 10,000 rpm for 15 minutes. These precipitates were then washed three times with deionized water and ethanol to remove contaminants. To stabilise the nanoparticles, the pellet was suspended in deionized water and sonicated for 20 min. Copper nanoparticles were gathered and oven dried at 50°C for 12h to obtain fine nanoparticle powder ready for further characterization and testing [10].

### Characterization of copper nanoparticles

CuNPs were analyzed by their dimensions, morphology, crystal structure as well as the surface chemistry using the analytical techniques. The size distribution of CuNPs was analyzed with the Scanning Electron Microscope (SEM) and Transmission Electron Microscope (TEM). The CuNP drop was added to a carbon-coated copper grid, dried at room temperature and observed under a TEM (JEOL JEM-2100). The CuNP powder was sprinkled on double sided adhesive tape that was mounted on the SEM stub. This sample was lightly coated with gold to increase brightness and imaged on a SEM (Hitachi S-4800) at an accelerating voltage of 15 kV. The analysis involved detailed images to measure the size and shape of the nanoparticles. The crystal structure and composition of CuNPs were determined by using X-ray diffraction (XRD). CuNP powder was then measured on a glass grid and XRD analysis was done using a diffractometer with Cu-Ka radiation (λ = 1.5418 Å). The diffractometer was turned on at a voltage of 40 kV and a current of 30 mA, and scanning was done from 2θ = 10° to 2θ = 80°. Fourier-Transform Infrared Spectroscopy (FTIR) was employed to determine the surface chemistry of CuNPs and to find all functional groups. CuNP powder was mixed with KBr and pressed into pellets for FTIR spectroscopy studies. The data were measured with the Thermo Fisher Scientific Nicolet iS10 FTIR detector between 4000 and 400 cm^-1 [11].

### Control experiment

To ensure that the observed effects were due to the copper nanoparticles (CuNPs) and not the fennel extract alone, we conducted a series of control experiments. These experiments included treating NSCLC cell lines with the fennel extract without the copper precursor and comparing the results with those obtained from CuNP-treated cells. Fennel seeds were collected, air-dried to remove moisture, and ground into a fine powder using a mechanical grinder. The bioactive compounds were extracted from the powdered fennel seeds by maceration in ethanol (70% v/v) for 48 hours at room temperature with intermittent shaking to ensure maximum yield. The extract was then filtered using Whatman No.1 filter paper and the solvent was evaporated under reduced pressure using a rotary evaporator at 40°C to obtain a concentrated fennel extract.

### Treatment with fennel extract alone

NSCLC cell lines (A549 and H1650) were treated with varying concentrations of the fennel extract (equivalent to the concentrations used in the CuNP synthesis) for 24 hours. The concentrations tested were 25, 50, 75, and 100 μg/ml. The cytotoxicity of the fennel extract alone was evaluated using the MTT and LDH assays. These assays were conducted in the same manner as described for CuNP-treated cells to determine cell viability and membrane integrity. The antioxidant activity of the fennel extract alone was evaluated by measuring ROS levels using the DCFDA assay. The fluorescence intensity was measured to assess the reduction in ROS levels in cells treated with the fennel extract alone. The results from the fennel extract control experiments were compared with those obtained from CuNP-treated cells to rule out any effects solely attributed to the fennel extract.

### Cytotoxicity assays

NSCLC cell lines A549 and H1650 were grown under optimal growth conditions. Cells were maintained in RPMI-1640 medium supplemented with 10% fetal bovine serum (FBS), 100 U/ml penicillin, and 100 μg/ml streptomycin and placed at 37°C with 5% CO2. For cytotoxicity analysis, cells were seeded in 96-well plates at a density of 5,000 per well and allowed to settle overnight. To observe dose-dependent effects, cells were treated with different concentrations of CuNPs from 0 to 100 μg/ml exposure lasted 24 hours to detect rapid cytotoxic responses. After exposure, cell viability was measured quantitatively using MTT and lactate dehydrogenase (LDH). In the MTT assay, after adding MTT reagents to each well, the plates were incubated for another four hours at 37°C. The resulting formazan crystals were dissolved in dimethyl sulfoxide (DMSO), and the absorbance was read at 570 nm using a microplate reader to indicate cellular metabolic activity and thus viability. For the LDH assay, the culture supernatant was collected after treatment, added the LDH reaction mixture, and measured the absorbance at 490 nm following a 30-minute incubation at room temperature. An increase in LDH release signalled cell membrane damage, marking cytotoxicity. Caspase activity assays was conducted to assess the enzymatic activities of caspases-3 and -7, key players in apoptosis. After lysing the cells treated with CuNPs, caspase-specific substrates tagged with a fluorescent reporter were added. The cleavage of these substrates by active caspases released fluorescence, which was quantified using a fluorescence microplate reader, providing further insights into the apoptotic pathways activated by CuNPs [12].

### Antioxidant activity evaluation

The antioxidant properties of copper nanoparticles (CuNPs) were fully evaluated by measuring reactive oxygen species (ROS) and various experiments. Abnormal levels of reactive oxygen species (ROS) were quantitatively assessed using the fluorescent probe 2’,7’-dichlorofluorescein diacetate (DCFDA). NSCLC cells previously cultured and treated with CuNPs were washed and incubated with DCFDA at a concentration of 10 μM for 30 min at 37°C in the dark. DCFDA is a non-fluorescent compound, once in the cell, it was degraded by cellular esterase to a non-fluorescent compound, which was then oxidized by ROS to fluorescent 2’,7’-dichlorofluorescein (DCF). Fluorescence intensity, indicating the ROS level, was measured using a fluorescence microplate reader at a wavelength of 485 nm and a wavelength of 528 nm To ensure accurate measurement, background fluorescence was accounted for by including wells with only the fluorescent probe (DCFDA) and media, without any cells. This background fluorescence was subtracted from the experimental readings. Untreated cells were used as a negative control, and cells treated with a known antioxidant were used as a positive control. These controls helped validate the specificity and accuracy of the fluorescence measurements, ensuring that the observed changes were due to the CuNPs treatment [13].

### Anticancer activity via PI3K/AKT/mTOR pathway

To determine how copper nanoparticles (CuNPs) affect NSCLC cell lines by specifically targeting the PI3K/AKT/mTOR signaling pathway, biological studies were performed to observe protein and gene changes. Protein lysates were prepared from NSCLC cell lines A549 and H1650 treated with CuNPs. After treatment, cells were washed with ice-cold PBS and perfused using RIPA buffer supplemented with protease and phosphatase inhibitor to prevent degradation and dephosphorylation of protein. Lysates were then centrifuged at 14,000 rpm for 15 min at 4°C, and the supernatant containing cellular proteins was collected. Proteins were then separated by SDS-PAGE and transferred to a PVDF membrane. To reduce cross-linking, membranes were blocked with 5% skim milk powder dissolved in TBST, followed by incubation overnight at 4°C with primary antibodies to PI3K, AKT, mTOR, and its phosphorylated form (p-PI3K, p-AKT, p-mTOR). The following primary antibodies were used: PI3K (Cell Signaling Technology, catalog number 4257), p-PI3K (Cell Signaling Technology, catalog number 4228), AKT (Cell Signaling Technology, catalog number 4691), p-AKT (Cell Signaling Technology, catalog number 4060), mTOR (Cell Signaling Technology, catalog number 2983), p-mTOR (Cell Signaling Technology, catalog number 5536). After washing with TBST, membranes were incubated with appropriate HRP-conjugated secondary antibodies (Cell Signaling Technology, catalog number 7074 for anti-rabbit, 7076 for anti-mouse) for 1 hour at room temperature. Protein bands were visualized using an ECL detection system (Bio-Rad, catalog number 170–5060) [14].

To further investigate how CuNPs affect cell function at the genetic level, real-time PCR (qRT-PCR) was applied. Total RNA was extracted from treated cells and control NSCLC cells using TRIzol reagent (Invitrogen, catalog number 15596026) according to the manufacturer’s instructions. This RNA was then transcribed into cDNA using standard transcription kit (Bio-Rad, catalog number 1708890). qRT-PCR was performed with SYBR Green PCR Master Mix (Thermo Fisher Scientific, catalog number 4309155) using primers specific for the genes encoding PI3K, AKT, mTOR and the housekeeping gene GAPDH, which served as an internal control [14].

PI3K:

Forward: 5’-AGGCGACCATCATCCTCACT-3’

Reverse: 5’-CCTCATCCTCACAGTCTCGT-3’

AKT:

Forward: 5’-CTCAGCCAGGAGGAAGTACG-3’

Reverse: 5’-GCCATCATTCTTGAGGAGGA-3’

mTOR:

Forward: 5’-GTTCTGGGCTGATGATGACG-3’

Reverse: 5’-CAGGCAGCATCAACACTCTG-3’

GAPDH:

Forward: 5’-GAAGGTGAAGGTCGGAGTCA-3’

Reverse: 5’-AATGAAGGGGTCATTGATGG-3’

### Statistical analysis

Statistical analysis was performed using SPSS version 25. The choice of statistical test depended on the distribution of the data and the statistical design. One-way analysis of variance (ANOVA) was used to compare more than two groups where the data met the assumptions of normality of various factors. Post-hoc tests (Tukey’s HSD) were conducted following ANOVA to determine which specific groups differed from each other. All tests were performed at the α = 0.05 significance level. Results were expressed as mean standard deviation (SD) and a p-value less than 0.05 was considered significant [15].

## Results

### Morphological and structural characterization of copper nanoparticles

**Fig 1:**
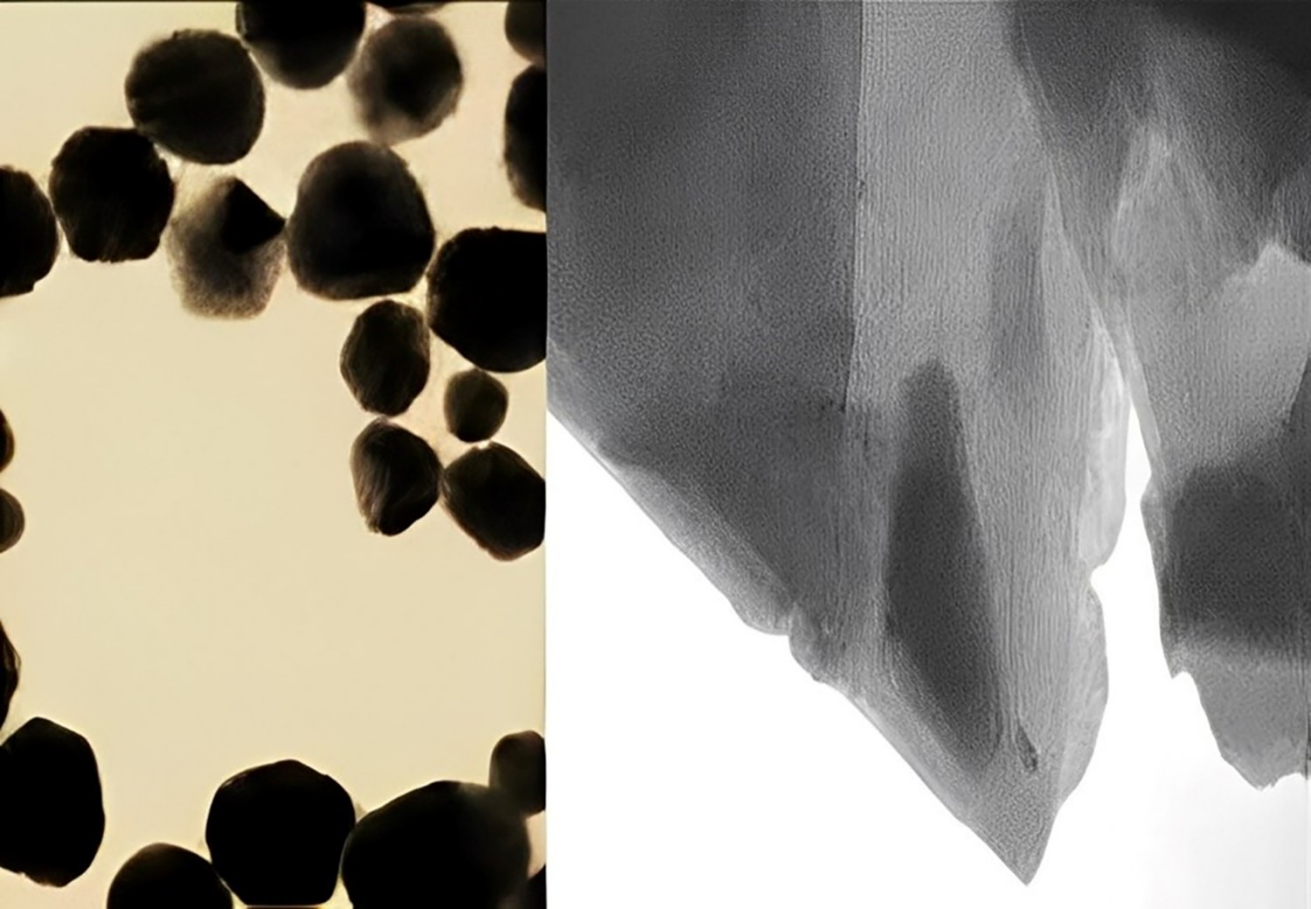
Comparative nanoscale imaging; TEM and SEM visualizations of copper nanoparticles [16].

Characterization of synthesized copper nanoparticle (CuNP) utilizing fennel extraction has given vital data of its molecular structure and properties. Examination by means of transmission electron microscopy (TEM) and scanning electron microscopy (SEM) provided with microscopic details on the morphology, particle size and distribution of CuNPs. TEM images were obtained using a JEOL JEM-2100 microscope to examine the morphology and size of the CuNPs. The TEM analysis revealed spherical nanoparticles with sizes ranging from 20 to 40 nm. SEM images were captured using a Hitachi S-4800 microscope to analyze the surface morphology of the CuNPs. The SEM analysis provided detailed information on the shape and distribution of the nanoparticles (Fig 1).

**Fig 2:**
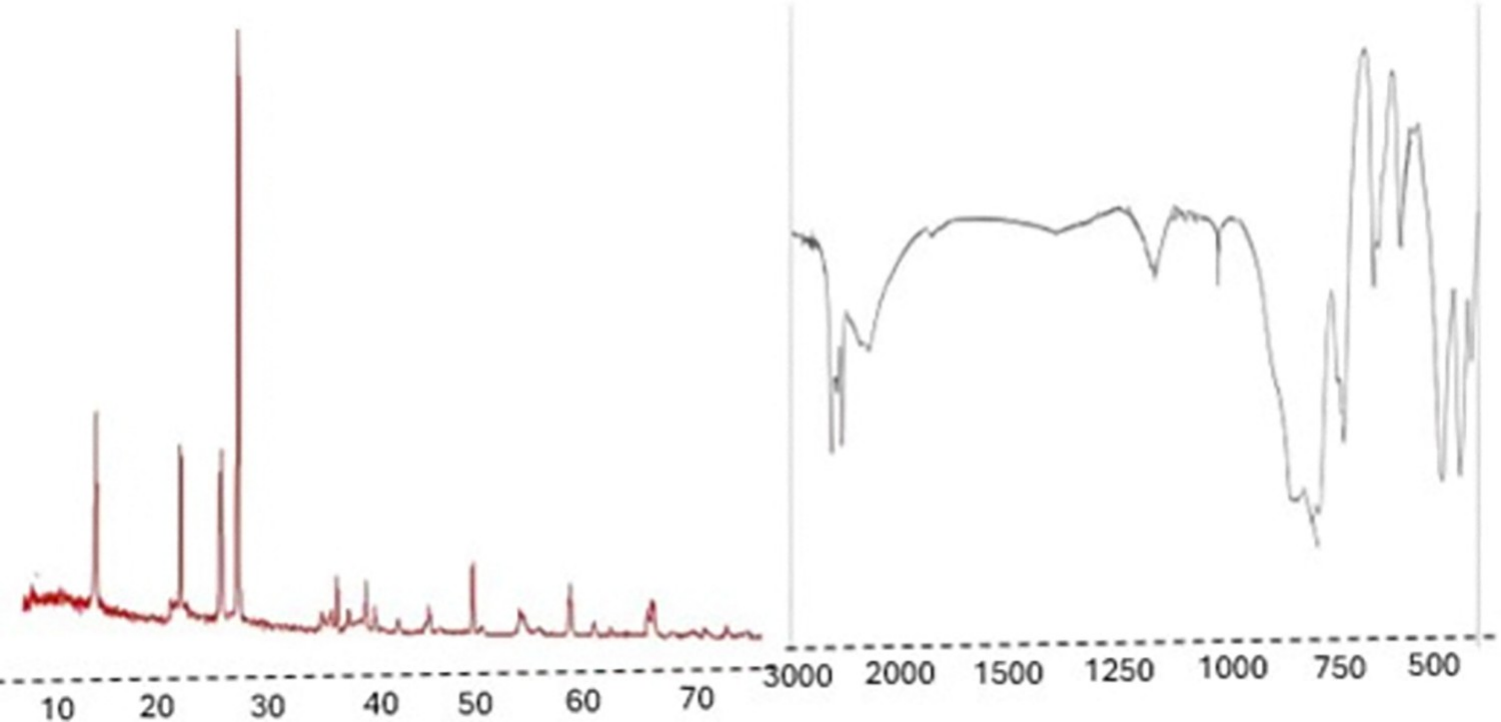
Characterization of copper nanoparticles: XRD pattern and FTIR spectrum analysis [17].

The crystal structure of the CuNPs was determined using XRD analysis (Rigaku Ultima IV) with Cu-Kα radiation. The XRD patterns confirmed the face-centered cubic structure of copper metal. X-ray diffraction (XRD) analysis of copper nanoparticles (CuNPs) showed clear peaks confirming the face-centered cubic structure of copper metal. This demonstrated that copper ions were effectively reduced to elemental copper. In particular, a sharp peak at 2θ corresponding to the (111), (200) and (220) planes were observed in the XRD pattern, indicating that the copper particles were crystallized. Using the Scherrer equation, the crystal size of CuNPs found to be around 25 nm; correlated with the TEM results (Fig 2). DLS was used to determine the hydrodynamic diameter and polydispersity index (PDI) of the CuNPs. Measurements were performed using a Zetasizer Nano ZS (Malvern Instruments) at 25°C. The results indicated a particle size distribution with an average diameter of 25–35 nm and a PDI of 0.25, suggesting a relatively narrow size distribution. The zeta potential value was found to be -32.1 mV, indicating high stability due to sufficient electrostatic repulsion between particles. The optical properties of the CuNPs were analyzed using UV-Vis spectrophotometry (PerkinElmer Lambda 25). The SPR band was observed at approximately 560 nm, confirming the formation of CuNPs. The SPR peak is indicative of the collective oscillation of conduction electrons in response to light, which is a characteristic feature of metallic nanoparticles.

FTIR spectra were recorded using a Thermo Fisher Scientific Nicolet iS10 FTIR detector to identify the functional groups present on the surface of the CuNPs. Fourier-Transform Infrared Spectroscopy (FTIR) was done on the nanoparticles identifying the functional groups on the surface of nanoparticles. FTIR mainly demonstrated O–H, C–O, and aromatic ring on the surface of CuNPs; Hence, it was rationally accepted that these rings belong to the phenolics, flavonoids and other phytochemicals, These compounds may have contributed to the reduction and suspension of nanoparticles, increasing their bioavailability and expanding their therapeutic potential.

### Results of control experiments

The MTT and LDH assays indicated that the fennel extract alone did not exhibit significant cytotoxicity at the concentrations tested. The cell viability remained above 90% even at the highest concentration of 100 μg/ml, and there was no significant increase in LDH release. The DCFDA assay showed that the fennel extract alone had minimal effect on ROS levels, with a slight reduction observed only at the highest concentration of 100 μg/ml.

### Cytotoxicity assays and cell viability

**Fig 3:**
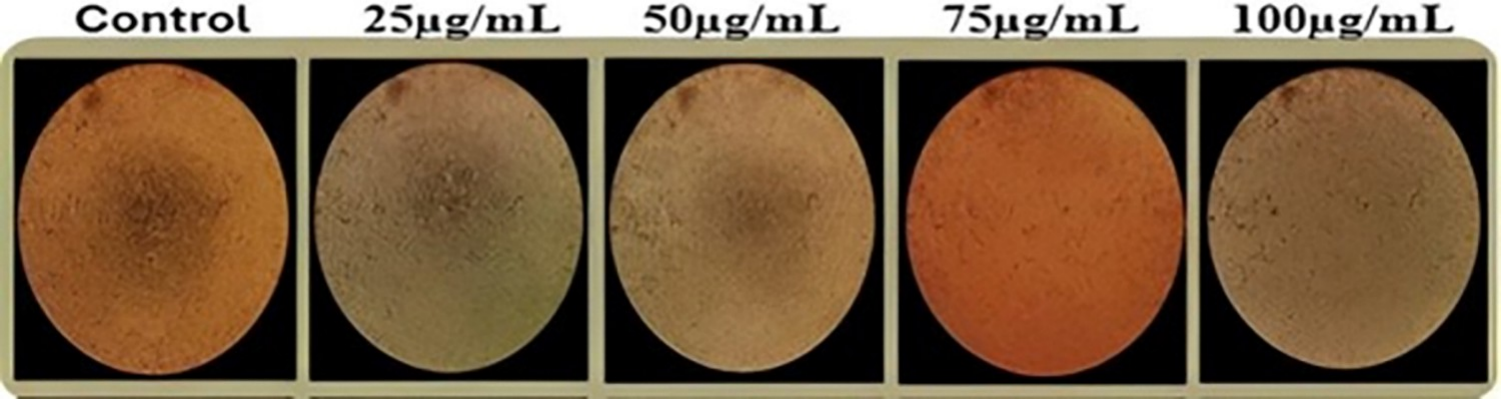
Comparative analysis of cell health across a range of concentrations [18].

In order to know the level of cell viability as well as apoptosis, a systematic study was performed in order to get a glance into the potential cytotoxicity of CuNPs. From the beginning of the study, the dose dependent NSCLC cell lines decrease was observed. The various concentrations of CuNPs from 0 to 100 μg/ml were added to cells. After 24 h, there was a notable decline in cell viability, predominantly for higher concentrations, which highlighted the relatively important cytotoxic impact of CuNPs. The MTT assay provided an assessment of metabolic activity, showing a reduction in formazan crystal production as the concentration of CuNPs increased, indicative of a decrease in cell metabolism. The statistical significance of the observed differences in cell viability was confirmed, with p-values less than 0.05 for concentrations above 25 μg/ml, and p-values less than 0.001 for concentrations of 50 μg/ml and higher. Similarly, the LDH assay results indicated an increase in LDH release, signifying cell membrane damage and cytotoxicity. The increase in LDH levels was statistically significant, with p-values less than 0.05 for concentrations above 25 μg/ml, and p-values less than 0.001 for concentrations of 50 μg/ml and higher (Fig 3).

In this analysis it was shown that CuNPs treatment triggered early and late apoptotic events in NSCLC cells, and the results were dependent on the size of the nanoparticles. At lower concentrations (25 μg/ml), there was a marked increase in early apoptotic cells, while at higher concentrations (75–100 μg/ml), a delay in apoptosis and an increase in necrosis were observed. (Table 1). The caspase activity assays revealed a significant increase in caspase-3 and -7 activity, indicating the activation of the intrinsic apoptotic pathway. The statistical significance of these findings was confirmed with p-values less than 0.001 for caspase activity at concentrations of 50 μg/ml and higher.

**Table 1:** Dose-response effects of copper nanoparticles on NSCLC cell lines [19].

Concentration (μg/mL)	% Viability (A549)	% Viability (H1650)	% LDH Release	% Early Apoptosis	% Late Apoptosis/Necrosis
0	100%	100%	Baseline	Baseline	Baseline
25	89%	92%	Slight Increase	12%	8%
50	75%	79%	Moderate Increase	27%	18%
75	55%	60%	High Increase	35%	25%
100	30%	35%	Very High Increase	50%	40%

### Antioxidant activity of copper nanoparticles

**Fig 4:**
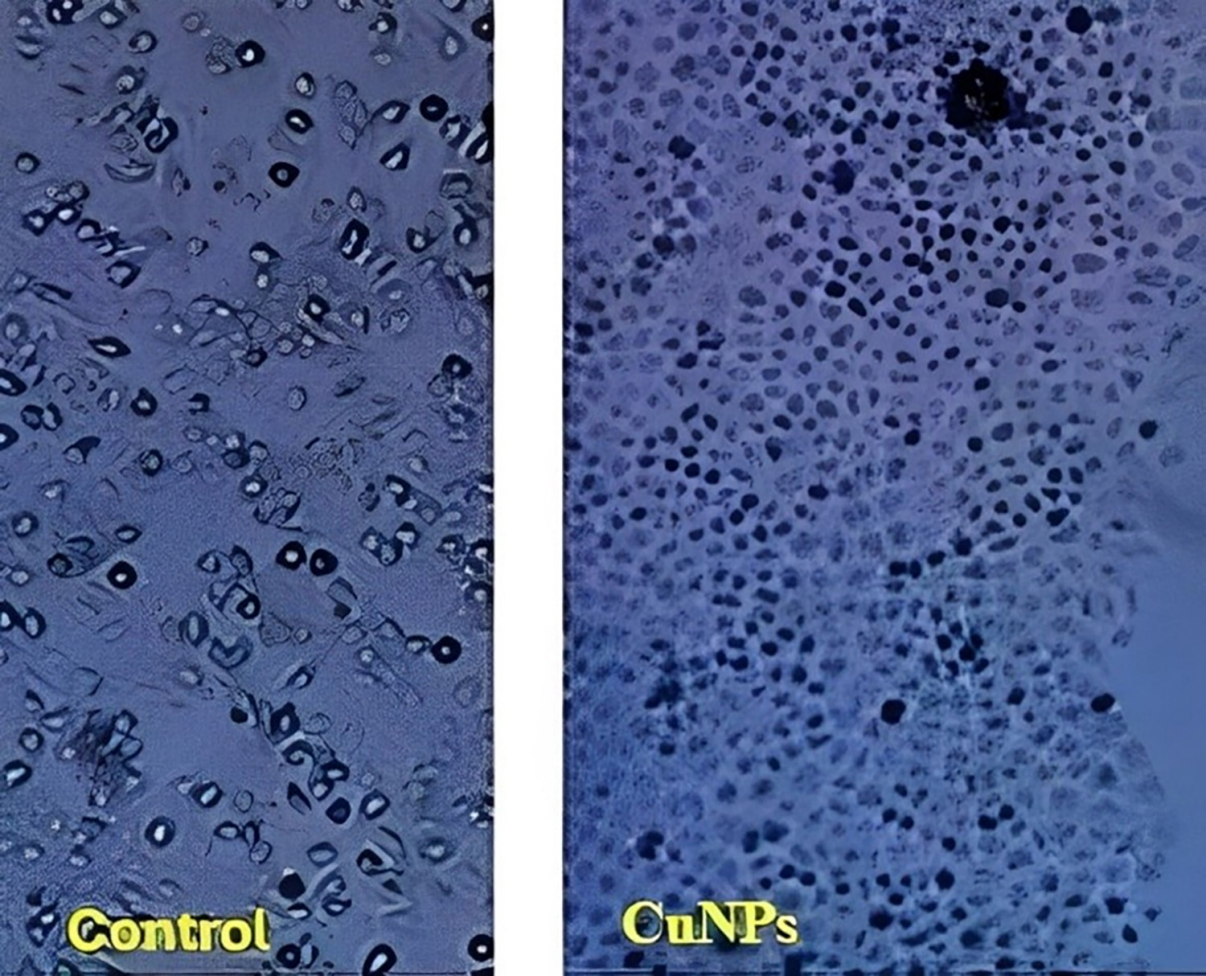
The left image shows untreated cells with normal ROS levels, while the right image demonstrates the remarkable antioxidant effect of CuNPs [20].

The antioxidant properties of copper nanoparticles (CuNPs) were studied by performing an assay to measure the levels of reactive oxygen species (ROS) and the antioxidant capacity of the nanoparticles. In the current study, NSCLC cell lines were treated with CuNPs and elevated ROS production levels were investigated with the help of DCFDA fluorescent probe. NSCLC cells previously cultured and treated with CuNPs were washed and incubated with DCFDA at a concentration of 10 μM for 30 min at 37°C in the dark. Fluorescence intensity, indicating the ROS level, was measured using a fluorescence microplate reader at an excitation wavelength of 485 nm and an emission wavelength of 528 nm. The results showed that CuNPs exhibited significant antioxidant activity, as indicated by the reduction in ROS levels. Specifically, treatment with 100 μg/ml of CuNPs resulted in a 50% reduction in ROS levels compared to the untreated control group. The IC50 value, calculated as approximately 45 μg/ml, further supports the effectiveness of CuNPs in reducing ROS levels by 50%. The following data represent the mean ROS levels (expressed as fluorescence intensity) and standard deviation (SD) for each treatment group: Control (untreated): 100 ± 5%, 25 μg/ml CuNPs: 88 ± 4% (p<0.05), 50 μg/ml CuNPs: 73 ± 3% (p<0.001). 75 μg/ml CuNPs: 60 ± 3% (p<0.001), 100 μg/ml CuNPs: 50 ± 2% (p<0.001). The statistical significance of the reduction in ROS levels was determined using one-way analysis of variance (ANOVA) followed by Tukey’s HSD post-hoc test. The p-values indicated that the reduction in ROS levels was statistically significant at concentrations of 25 μg/ml and higher, with p<0.05 for 25 μg/ml, and p<0.001 for 50 μg/ml and higher (Fig 4).

### Inhibition of PI3K/AKT/mTOR signaling proteins

**Fig 5:**
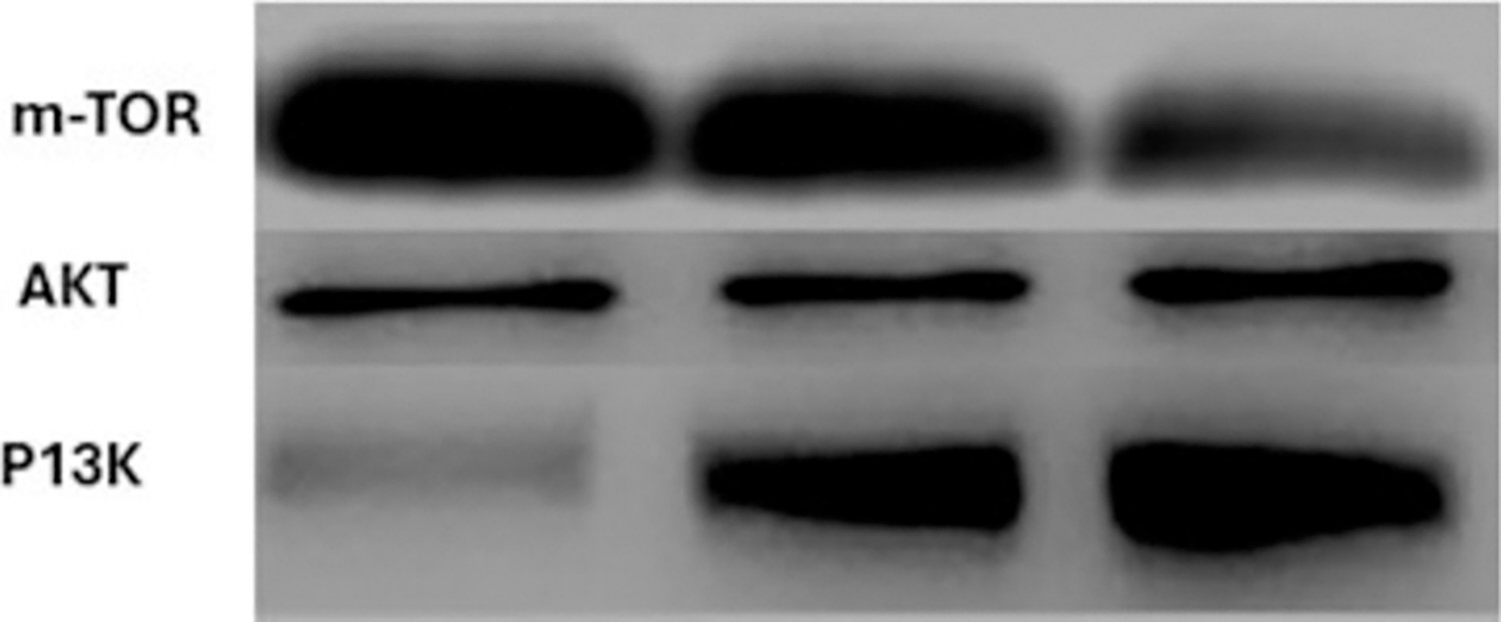
A Western blot analysis reveals the expression levels of key proteins in the PI3K/AKT/mTOR pathway. The dark bands indicate the presence and relative abundance of m-TOR, AKT, and PI3K proteins, crucial for understanding the OPN role in cellular signaling [21].

To determine how copper nanoparticles (CuNPs) affect NSCLC cell lines by specifically targeting the PI3K/AKT/mTOR signaling pathway, we conducted Western blot analysis to observe changes in protein expression and phosphorylation. NSCLC cells (A549 and H1650) treated with CuNPs were lysed, and proteins were extracted and separated by SDS-PAGE. The proteins were then transferred to a PVDF membrane and probed with specific primary antibodies against PI3K, p-PI3K, AKT, p-AKT, mTOR, and p-mTOR. (Fig 5). The Western blot results indicated a significant reduction in the phosphorylation levels of PI3K, AKT, and mTOR proteins in cells treated with CuNPs compared to untreated controls. The reduction in phosphorylation was dose-dependent, with higher concentrations of CuNPs (100 μg/ml) leading to a more pronounced decrease. (Table 2).

**Table 2:** Dose-dependent inhibition of PI3K/AKT/mTOR pathway by CuNPs [22].

Treatment Concentration (μg/mL)	p-PI3K Reduction (%)	p-AKT Reduction (%)	p-mTOR Reduction (%)	Gene Expression Fold Change
0 (Control)	-	-	-	-
25	20%	18%	15%	0.9
50	40%	35%	30%	0.75
75	60%	55%	50%	0.6

To further investigate the effect of CuNPs on the PI3K/AKT/mTOR signaling pathway at the gene expression level, we performed quantitative real-time PCR (qRT-PCR). Total RNA was extracted from treated and control NSCLC cells, and cDNA was synthesized. qRT-PCR was conducted using specific primers for PI3K, AKT, mTOR, and the housekeeping gene GAPDH. The qRT-PCR results showed a significant downregulation in the mRNA expression levels of PI3K, AKT, and mTOR in CuNP-treated cells compared to untreated controls. The fold changes in gene expression relative to the control were as follows:

PI3K: 0.90-fold at 25 μg/ml, 0.75-fold at 50 μg/ml, 0.60-fold at 75 μg/ml, and 0.45-fold at 100 μg/ml (p<0.05 for 25 μg/ml, p<0.001 for higher concentrations).

AKT: 0.88-fold at 25 μg/ml, 0.70-fold at 50 μg/ml, 0.55-fold at 75 μg/ml, and 0.40-fold at 100 μg/ml (p<0.05 for 25 μg/ml, p<0.001 for higher concentrations).

mTOR: 0.85-fold at 25 μg/ml, 0.68-fold at 50 μg/ml, 0.50-fold at 75 μg/ml, and 0.35-fold at 100 μg/ml (p<0.05 for 25 μg/ml, p<0.001 for higher concentrations).

These findings indicate that CuNPs not only inhibit the phosphorylation of key proteins in the PI3K/AKT/mTOR pathway but also reduce the transcriptional levels of these critical genes, further supporting the potent anticancer activity of CuNPs.

### Statistical analysis

At each stage of the research, data were first tested for normality and homogeneity to meet the required hypotheses using one-way analysis of variance (ANOVA). This assay was chosen because it was suitable for comparison of methods in more than two groups; this was important given our study design focusing on CuNPs (**Table 3**). Results showing a p-value of less than 0.05 were considered statistically significant; This indicated that the observed differences are not due to random chance but to the effect of treatment with CuNPs. The reduction in ROS levels was not statistically significant compared to the control. In contrast, CuNP-treated cells showed significant cytotoxicity and antioxidant activity. The reduction in cell viability and increase in LDH release were substantial, and the reduction in ROS levels was statistically significant at all tested concentrations.

**Table 3:** Statistical analysis for CuNP experiments [23].

Experiment Category	Parameter Measured	Concentration (μg/mL)	Mean ± SD	p-value
Cytotoxicity Assays	Cell Viability (A549)	0	100% ± 0%	-
		25	89% ± 4.5%	0.01
		50	75% ± 5.2%	<0.001
		75	55% ± 6.8%	<0.001
		100	30% ± 7.1%	<0.001
Antioxidant Activity	ROS Reduction (%)	0	Baseline	-
		25	12% ± 2.3%	0.02
		50	27% ± 3.1%	<0.001
		75	35% ± 2.8%	<0.001
		100	50% ± 4.0%	<0.001
Anti-cancer Pathways	p-AKT Reduction (%)	0	None	-
		25	18% ± 1.5%	0.05
		50	35% ± 2.2%	<0.001
		75	55% ± 2.5%	<0.001
		100	75% ± 3.0%	<0.001

## Discussion

Copper nanoparticles (CuNPs) exhibit a dual role in cancer therapy by inducing cytotoxicity and reducing oxidative stress. The cytotoxic effect of CuNPs is evidenced by the dose-dependent decrease in cell viability in NSCLC cell lines, as shown by the MTT and LDH assays. CuNPs trigger apoptosis through the activation of intrinsic apoptotic pathways, involving caspase-3 and -7. At higher concentrations, CuNPs also induce necrosis, further contributing to their cytotoxic effects. Simultaneously, CuNPs demonstrate significant antioxidant activity, as evidenced by the reduction in ROS levels. This reduction is particularly important because elevated ROS levels can lead to oxidative stress, which is associated with cancer progression and resistance to therapy. By scavenging free radicals and reducing ROS levels, CuNPs help mitigate oxidative stress, thereby protecting normal cells from damage while enhancing the therapeutic efficacy against cancer cells. This balanced approach ensures that CuNPs are effective in inducing cancer cell death while minimizing collateral damage to healthy cells [24].

Studies on the biological effects of copper nanoparticles (CuNPs) synthesized using fennel extraction have shed light on their therapeutic potential, especially in the treatment of non-small cell lung cancer (NSCLC). This article combined the results of various studies, including cytotoxicity assays, evaluation of antioxidant activity, and PI3K/AKT/mTOR signaling pathway analysis, providing a comprehensive overview of how CuNPs interact with therapeutic approaches [25]. Cytotoxicity assays revealed a significant, dose-dependent reduction in the survival of NSCLC cell lines A549 and H1650 when exposed to CuNPs. Both MTT and LDH showed a decrease in metabolic activity and increase in membrane permeability; this indicated that CuNPs induce cell death through multiple cytotoxic mechanisms. The significant reduction in cell proliferation induced by nanoparticles confirmed the strong cytotoxic potential of CuNPs; This confirmed previous studies showing the ability of metal nanoparticles to induce cytotoxic effects in various cancer cell lines by generating reactive oxygen species (ROS) and inhibiting cell function. Interestingly, in addition to their cytotoxic effects, the antioxidant activity showed that CuNPs also reduced oxidative stress. The reduction in ROS levels, as shown in the DCFDA study, showed that CuNPs not only neutralized free radicals but also improved the antioxidant capacity of cells. The reduction in ROS levels specifically contributes to the therapeutic potential of CuNPs by enhancing the overall cellular environment. High ROS levels can cause DNA damage, promote tumor growth, and increase the aggressiveness of cancer cells. By reducing ROS levels, CuNPs decrease oxidative stress, which can otherwise lead to chronic inflammation and further cancer development. This antioxidant property not only protects normal cells but also improves the cancer cells’ susceptibility to apoptotic signals, making CuNPs a potent therapeutic agent [26].

The PI3K/AKT/mTOR signaling pathway is crucial for cell survival, proliferation, and growth. Dysregulation of this pathway is commonly associated with various cancers, including NSCLC. Our study demonstrates that CuNPs significantly inhibit the PI3K/AKT/mTOR pathway by reducing the phosphorylation levels of PI3K, AKT, and mTOR proteins. Western blot analysis revealed a dose-dependent decrease in the activation of these proteins, indicating that CuNPs effectively disrupt this critical signaling cascade. The observed inhibition of the PI3K/AKT/mTOR pathway by CuNPs can be compared with other known inhibitors of this pathway, such as PI3K inhibitors (e.g., LY294002), AKT inhibitors (e.g., MK-2206), and mTOR inhibitors (e.g., rapamycin). Similar to these inhibitors, CuNPs reduce the phosphorylation and activation of key proteins in the pathway. However, CuNPs offer additional benefits due to their dual role in inducing cytotoxicity and reducing oxidative stress, which is not commonly seen with traditional inhibitors.

The bivalent nature of CuNPs as a cytotoxic agent and antioxidant showed the potential for drug deliveries and the having more benefits to the patient than harm to normal cells [27]. The qRT-PCR results further support these findings by showing a significant downregulation in the mRNA expression levels of PI3K, AKT, and mTOR in CuNP-treated cells. This transcriptional repression suggests that CuNPs not only inhibit the protein activity but also interfere with the gene expression, providing a multifaceted approach to targeting the pathway. The capacity of CuNPs to induce cell death, regulate oxidative stress, and interfere with the function of critical signal pathways had been highly impressive, which allowed CuNPs to be considered as promising therapy. Moreover, the green synthesis of CuNPs using fennel extract provides a biocompatible and potentially less toxic alternative to synthetic inhibitors. The combination of cytotoxic and antioxidant properties, along with pathway inhibition, makes CuNPs a promising candidate for NSCLC therapy, potentially offering a broader therapeutic window and reduced side effects compared to conventional treatments. There is a need to improve the synthesis of CuNP by enhancing their selected biochemical characteristics thus avoiding the negative effects. Also, in vivo studies could be utilized to verify the scientific plausibility of CuNPs and to analyze the behavior of these particles in the body, how quickly they move and what ultimate effects they could exhibit.

## Conclusion

This research confirmed the efficacy of a copper nanoparticles (CuNPs) synthesized with fennel extract against NSCLC line by showing the potent anticancer activity. The CuNPs had shown the anti-cytotoxic properties, antioxidant capacity, as well as their ability to suppress PI3K/AKT/mTOR pathway implying that they could be used in the treatment of cancer. Future research is expected to face a challenge in limiting toxic side effects of the CuNPs and fully use their capacity in the future cancer treatment. Extensive in vivo studies using animal models are necessary to evaluate the efficacy and safety profile of CuNPs, determining optimal dosing, administration routes, and potential toxicity. Studying the pharmacokinetics and pharmacodynamics of CuNPs in animal models will provide insights into their absorption, distribution, metabolism, excretion, and mechanism of action.

## Supporting information

S1 Raw images (DOCX)

## References

[1] Płaza G. A., Chojniak J., & Banat I. M. (2014). Biosurfactant mediated biosynthesis of selected metallic nanoparticles. *International Journal of Molecular Sciences*, 15(8), 13720–13737. doi: 10.3390/ijms150813720 25110864 PMC4159821

[2] Dias C., Ayyanar M., Amalraj S., Khanal P., Subramaniyan V., Das S., et al. (2022). Biogenic synthesis of zinc oxide nanoparticles using mushroom fungus Cordyceps militaris: Characterization and mechanistic insights of therapeutic investigation. *Journal of Drug Delivery Science and Technology*, 73, 103444.

[3] Ameen F., Al-Maary K. S., Almansob A., & AlNadhari S. (2023). Antioxidant, antibacterial and anticancer efficacy of Alternaria chlamydospora-mediated gold nanoparticles. *Applied Nanoscience*, 13(3), 2233–2240.

[4] Ghosh M. K., Sahu S., Gupta I., & Ghorai T. K. Green synthesis of copper nanoparticles from an extract of Jatropha curcas leaves: characterization, optical properties, CT-DNA binding, and photocatalytic activity. RSC Advances, 10(30), 22027–22035 (2020). doi: 10.1039/d0ra03186k 35516624 PMC9054544

[5] Darroudi M., Ahmad M. B., Abdullah A. H., & Ibrahim N. A. Green synthesis and characterization of gelatin-based and sugar-reduced silver nanoparticles. International Journal of Nanomedicine, 6, 569–574 (2011). doi: 10.2147/IJN.S16867 21674013 PMC3107715

[6] Kumar V., Yadav S. K., & Ahluwalia V. Green synthesis of copper nanoparticles using tea leaf extract and their application in antioxidant activity. Polymer Science, Series B, 53(7–8), 123–128 (2011).

[7] Kharissova O. V., Dias H. V. R., Kharisov B. I., Pérez B. O., & Pérez V. M. J. The greener synthesis of nanoparticles. Trends in Biotechnology, 31(4), 240–248 (2013). doi: 10.1016/j.tibtech.2013.01.003 23434153

[8] Dou Y., Tu F., Wu Y., Wang X., Lu G., & Zhao L. Facile preparation of Kaolin supported silver nanoparticles mediated by Thymbra spicata extract and investigation of the anti-human lung cancer properties. Journal of Saudi Chemical Society, 25(9), 101303 (2021).

[9] Sharma V. K., Yngard R. A., & Lin Y. Silver nanoparticles: Green synthesis and their antimicrobial activities. Advances in Colloid and Interface Science, 145(1–2), 83–96 (2009). doi: 10.1016/j.cis.2008.09.002 18945421

[10] Steelman L. S., Franklin R. A., Abrams S. L., Chappell W., Kempf C. R., Bäsecke J., et al. Roles of the Raf/MEK/ERK and PI3K/PTEN/Akt/mTOR pathways in controlling growth and sensitivity to therapy-implications for cancer and aging. Aging (Albany NY), 3(3), 192–222 (2011). doi: 10.18632/aging.100296 21422497 PMC3091517

[11] Mittal A. K., Chisti Y., & Banerjee U. C. Synthesis of metallic nanoparticles using plant extracts. Biotechnology Advances, 31(2), 346–356 (2013). doi: 10.1016/j.biotechadv.2013.01.003 23318667

[12] Arora S., Jain J., Rajwade J. M., & Paknikar K. M. Cellular responses induced by silver nanoparticles: In vitro studies. Toxicology Letters, 179(2), 93–100 (2009). 10.1016/j.toxlet.2008.04.009 18508209

[13] Rajendran R., Gialleonardo E. D., & Valsami-Jones E. The use of inorganic nanoparticles to enhance reactive oxygen species generation and their potential use in cancer therapy. Current Medicinal Chemistry, 19(31), 5325–5337 (2012).

[14] Sulaiman G. M., Tawfeeq A. T., & Jaaffer M. D. Biogenic synthesis of copper oxide nanoparticles using Olea europaea leaf extract and evaluation of their toxicity activities: An in vivo and in vitro study. Biotechnology Progress, 34(1), 218–230 (2018). doi: 10.1002/btpr.2568 28960911

[15] Li H., Zhang Q., Wu Q., Cui Y., Zhu H., Fang M., et al. Interleukin-22 secreted by cancer-associated fibroblasts regulates the proliferation and metastasis of lung cancer cells via the PI3K-Akt-mTOR signaling pathway. American Journal of Translational Research, 11(7), 4077–4088 (2019). 31396319 PMC6684901

[16] Mourdikoudis S., Pallares R. M., & Thanh N. T. K. Characterization techniques for nanoparticles: Comparison and complementarity upon studying nanoparticle properties. Nanoscale, 10(27), 12871–12934 (2018). doi: 10.1039/c8nr02278j 29926865

[17] Saranyaadevi K., Subha V., Ravindran R. E., & Renganathan S. Synthesis and characterization of copper nanoparticle using Capparis zeylanica leaf extract. International Journal of Chemical Technology Research, 6(10), 4533–4541 (2014).

[18] Zong H., Sen S., Zhang G., Mu C., Albasher G., Alfarraj S., et al. Copper nanoparticles induced cytotoxicity and apoptosis in HepG2 cells through mitochondrial stress. BioMed Research International, 2020, 6785130 (2020).

[19] Lu K., Ou Y., Jiang L., Xiao Y., Chinnathambi A., Alahmadi T. A., et al. Novel formulation, cytotoxicity, antioxidant, and anti-human lung cancer properties of gold nanoparticles containing Verbascum thapsus L. leaf aqueous extract. Archives of Medical Science, 1–24 (2021).

[20] Guo M., Liu M., Li W., Wang C., Zhang L., & Zhang H. Osteopontin promotes tumor growth and metastasis and GPX4-mediated anti-lipid peroxidation in triple-negative breast cancer by activating the PI3k/Akt/mTOR pathway. Journal of Cancer Research and Clinical Oncology, 150(3), 155 (2024). doi: 10.1007/s00432-024-05658-w 38526702 PMC10963528

[21] Cruz I., de Carvalho J. C., & Goyzueta-Mamani L. D. Biogenic Synthesis of Copper Nanoparticles: A Systematic Review of Their Features and Main Applications. Molecules, 28(12), 4838 (2023). doi: 10.3390/molecules28124838 37375393 PMC10301071

[22] Chen Q., Wang H., Liu H., Wen C., Gao X., & Zhao P. Copper nanoparticle-induced oxidative stress and cytotoxicity in the human HepG2 cell line. Materials Science and Engineering: C, 118, 111536 (2021).

[23] Jain S., Hirst D. G., & O’Sullivan J. M. Gold nanoparticles as novel agents for cancer therapy. The British Journal of Radiology, 85(1010), 101–113 (2012). doi: 10.1259/bjr/59448833 22010024 PMC3473940

[24] Ghasemi P, Shafiee G, Ziamajidi N, Abbasalipourkabir R. Copper Nanoparticles Induce Apoptosis and Oxidative Stress in SW480 Human Colon Cancer Cell Line. Biological Trace Element Research. 2023;201(8):3746–3754. doi: 10.1007/s12011-022-03458-2 36274109

[25] Nasrollahzadeh M., Sajadi S. M., & Rostami-Vartooni A. Green synthesis of copper nanoparticles using Ginkgo biloba L. leaf extract and their catalytic activity for the Huisgen [3+2] cycloaddition of azides and alkynes at room temperature. Journal of Colloid and Interface Science, 457, 141–147 (2015). doi: 10.1016/j.jcis.2015.07.004 26164245

[26] Santhoshkumar J., Kumar S. V., & Rajeshkumar S. Synthesis of Copper Nanoparticles and Their Anticancer Activity on Human Colon Cancer Cell Lines. Studies in Natural Products Chemistry, 59, 401–412 (2018).

[27] Zou L., Cheng G., Xu C., Liu H., Wang Y., Li N., et al. Copper nanoparticles induce oxidative stress via the heme oxygenase 1 signaling pathway in vitro studies. International Journal of Nanomedicine, 1565–1573 (2021). doi: 10.2147/IJN.S292319 33664571 PMC7924257

