# Supplementary figures and images for: Characterization and evaluation of the cytotoxic, antioxidant, and anti-human lung cancer properties of copper nanoparticles green-synthesized by fennel extract following the PI3K/AKT/Mtor signaling pathway
Source: PLoS One. 2025 Jan 9;20(1):e0309207. doi: 10.1371/journal.pone.0309207 (PMC11717185; doi:10.1371/journal.pone.0309207)

Uncropped western blot


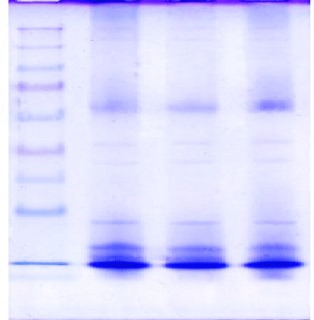

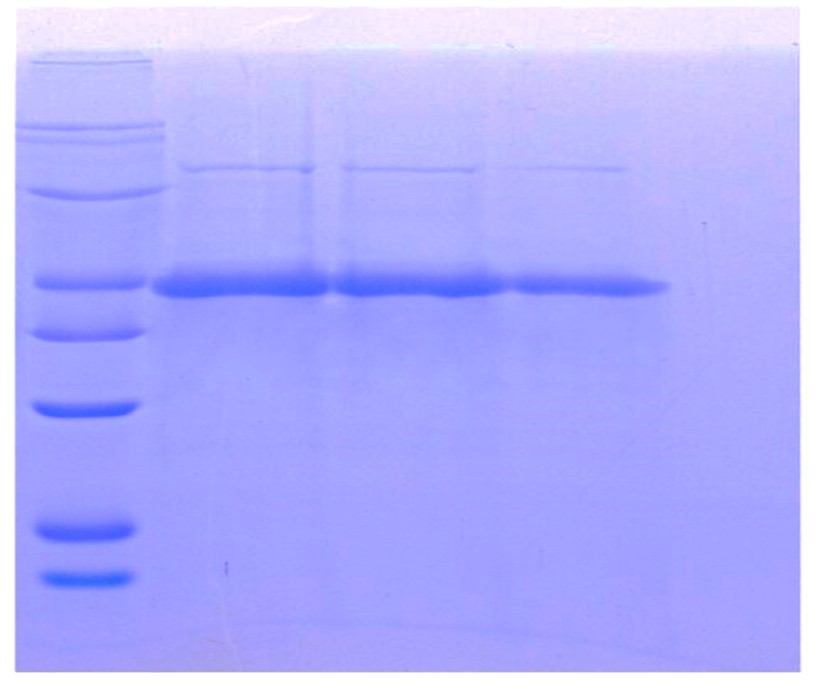

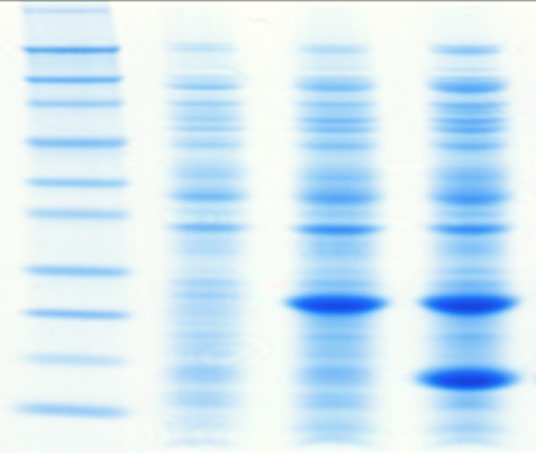

Supplement: S1 Raw images — (DOCX) [file pone.0309207.s001.docx]
